# Linking beaver dam affected flow dynamics to upstream passage of Arctic grayling

**DOI:** 10.1002/ece3.4728

**Published:** 2018-12-04

**Authors:** Kyle A. Cutting, Jake M. Ferguson, Michelle L. Anderson, Kristen Cook, Stacy C. Davis, Rebekah Levine

**Affiliations:** ^1^ Red Rock Lakes National Wildlife Refuge U.S. Fish and Wildlife Service Lima Montana; ^2^ Department of Fisheries, Wildlife & Conservation Biology University of Minnesota St. Paul Minnesota; ^3^ Department of Biology The University of Montana Western Dillon Montana; ^4^ Ennis National Fish Hatchery U.S. Fish and Wildlife Service Ennis Montana; ^5^ Department of Land Resources and Environmental Sciences Montana State University Bozeman Montana; ^6^ Department of Environmental Sciences The University of Montana Western Dillon Montana

**Keywords:** adaptive management, beaver dam, biodiversity, *Castor canadensis*, fish passage, *Thymallus arcticus*

## Abstract

Beaver reintroductions and beaver dam structures are an increasingly utilized ecological tool for rehabilitating degraded streams, yet beaver dams can potentially impact upstream fish migrations. We collected two years of data on Arctic grayling movement through a series of beaver dams in a low gradient mountain stream, utilizing radio‐telemetry techniques, to determine how hydrology, dam characteristics, and fish attributes impeded passage and movement rates of spawning grayling. We compared fish movement between a “normal” flow year and a “low” flow year, determined grayling passage probabilities over dams in relation to a suite of factors, and predicted daily movement rates in relation to the number of dams each fish passed and distance between dams during upstream migration to spawning areas. We found that the average passage probability over unbreached beaver dams was 88%, though we found that it fell below 50% at specific dams. Upstream passage of grayling was affected by three main characteristics: (a) temperature, (b) breach status, and (c) hydrologic linkages that connect sections of stream above and below the dam. Other variables influence passage, but to a lesser degree. Cumulative passage varied with distance upstream and total number of dams passed in low versus normal flow years, while movement rates upstream slowed as fish swam closer to dams. Our findings demonstrate that upstream passage of fish over beaver dams is strongly correlated with hydrologic conditions with moderate controls by dam‐ and fish‐level characteristics. Our results provide a framework that can be applied to reduce barrier effects when and where beaver dams pose a significant threat to the upstream migration of fish populations while maintaining the diverse ecological benefits of beaver activity when dams are not a threat to fish passage.

## 1 INTRODUCTION

**Figure 1:**
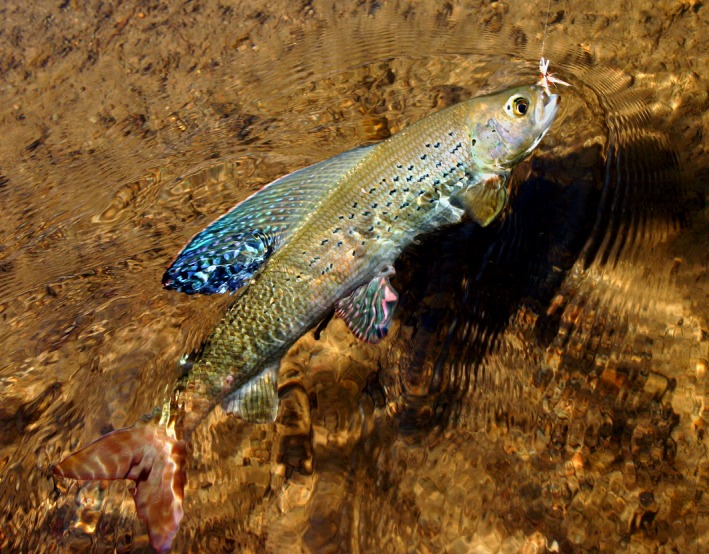
Photograph of adult Arctic grayling (*Thymallus arcticus*), Red Rock Creek, Red Rock Lakes National Wildlife Refuge, Montana, USA. Photograph taken by Grant Meyer, used with permission

Natural resource managers often note the difficulty of conserving multiple species and ecological processes in ecosystems, especially when species are perceived to have competing habitat requirements. One example of this phenomenon occurs in managing for migratory fish in landscapes extensively influenced by beavers. Beaver (*Castor *spp.) and grayling fish (*Thymallus* spp.) share an evolutionary history and Holarctic distribution shaped by glaciation over millions of years (Horn et al., 2011; Stamford & Taylor, 2004) and more recently by human impacts. Commercial overharvest nearly extirpated global beaver populations by the 1800s (Rosell, Bozser, Collen, & Parker, 2005), with profound consequences for stream hydrology, geomorphology, and biota across the Northern Hemisphere (Collen & Gibson, 2000; Naiman, Johnston, & Kelley, 1988; Pollock et al., 2014). A recent rebound in estimated numbers of Eurasian beaver (*Castor fiber*, >1 million; Halley, Roswell, & Saveljev, 2012) and North American beaver (*Castor canadensis*, 6–30 million; Naiman et al., 1988) resulted from natural recolonization and purposeful translocations. In contrast, European grayling (*Thymallus thymallus*) and Arctic grayling (*Thymallus arcticus*; Figure 1) have exhibited precipitous localized declines in alpine environments and along distributional edges (Northcote, 1995; Uiblein, Jagsch, Honsig‐Erlenburg, & Weiss, 2001), caused by heavy angling pressure (Northcote, 1995), competition with non‐native fish (Cutting, Cross, Anderson, & Reese, 2016), and loss of hydrologic connectivity and spawning and rearing habitat (Northcote, 1995). Southern populations of Arctic grayling in the contiguous United States were extirpated in the mid‐20th century from Michigan's Great Lakes region and were reduced to a fraction of their historic extent in Montana's Upper Missouri River basin (Peterson & Ardren, 2009). Due in part to evidence that grayling from the Upper Missouri provide a genetic resource of high conservation value (Peterson & Ardren, 2009), significant efforts are being directed toward recovering this population by targeting non‐native fish management and restoring in‐stream habitat conditions and connectivity.

Beaver reintroductions have been proposed as an ecological tool for restoring lost or degraded ecological processes related to aquatic habitat heterogeneity, channel sediment storage, and riparian forest structure (Burchsted, Daniels, Thorson, & Vokoun, 2010; Gurnell, 1998; Pollock et al., 2014), as well as mitigating negative effects of climate change on hyporheic exchange and variable in‐stream flows (Rosell et al., 2005). However, an affiliated area of particular interest and debate lies in demonstrating explicit linkages between beaver dam characteristics, streamflow dynamics, and fish movement kinetics as impacting upstream fish passage (Taylor, MacInnis, & Floyd, 2010), a subject warranting further research using empirical field studies coupled with modeling (Kemp, Worthington, Langford, Tree, & Gaywood, 2012; Lokteff, Roper, & Wheaton, 2013; Meixler, Baina, & Walter, 2009). Lokteff et al. (2013) found that native salmonids had higher passage frequency across beaver dams than did non‐native salmonid species, suggesting long‐term sympatry between native salmonids and beavers. However, few studies on potential barriers to Arctic grayling movement exist, and they focus primarily on culvert stream crossings (e.g., Travis & Tilsworth, 1986, Jones, Kiceniuk, & Bamford, 1974, C. MacPhee and F. J. Watts, unpublished data). In one instance, a 30.5‐m‐long and 1.5‐m‐wide culvert blocked passage of wild grayling until stream velocity receded to below 2.1 m/s (Travis & Tilsworth, 1986). In a controlled experimental setting, Jones et al. (1974) showed that individual grayling with the longest body lengths (40 cm) were capable of passing through a 100‐m‐long culvert in 10 min when velocities were less than 0.6 m/s.

In the recent past, restricted grayling passage and fragmentation in the upper Missouri basin likely had less of an effect on the population because grayling were widely distributed and abundant. Today, with the reduction in spatial extent and abundance of grayling in Montana (Peterson & Ardren, 2009), it is unclear whether beaver dams form localized barriers impeding fish passage to spawning habitat. Spawning movements of adfluvial grayling are synchronized annually with ice breakup and spring runoff (Northcote, 1995). While high flows likely allow grayling to more easily navigate across intact beaver dams enroute to upstream spawning reaches, passage may be compromised if spring runoff is low during the migration period. Impediments to passage may be exacerbated in northern latitudes if recent declines in accumulated snowpack and runoff timing persist (Kormos, Luce, Wenger, & Berghuijs, 2016).

Beaver dams have been considered significant barriers, in some years, to upstream migration by spawning grayling (Nelson, 1954), but empirical evidence to test this assumption is needed. Our study examined the effect of beaver dams on grayling movement and was designed to determine relationships for hydrology, dam features, and fish attributes that could impede fish passage and affect movement rates of spawning grayling. Given the dynamic nature of spring runoff in mountainous areas and potential energetic costs associated with migration through a heterogenous hydrogeomorphic landscape (e.g., McElroy, DeLonay, & Jacobson, 2012), we hypothesized that passage and movement of grayling are significantly influenced by environmental, dam, and fish characteristics. We predicted that barrier effects on upstream fish passage would be greater for unbreached versus breached dams. We also predicted that barrier events during snowmelt runoff are greater at lower flows relative to higher flows, both within and among years, which may reduce the ability of grayling to pass many dams and to reach upper basin spawning areas. We further predicted that time‐varying vertical jump height over dams, as jump height is considered to be a major factor influencing passage success or failure at barriers for a wide variety of salmonid species (Kondratieff & Myrick, 2006), would reduce passage probability across beaver dams. Finally, we predicted higher grayling movement velocity with greater distance between dams, but as the number of dams passed increases, movement velocity will decrease. In order to address these hypotheses and predictions, we conducted a 2‐year study of Arctic grayling spawning passage and movement utilizing radio‐telemetry techniques in a low gradient mountain stream with abundant beaver dams.

## 2 MATERIALS AND METHODS

### 2.1 Study area

**Figure 2:**
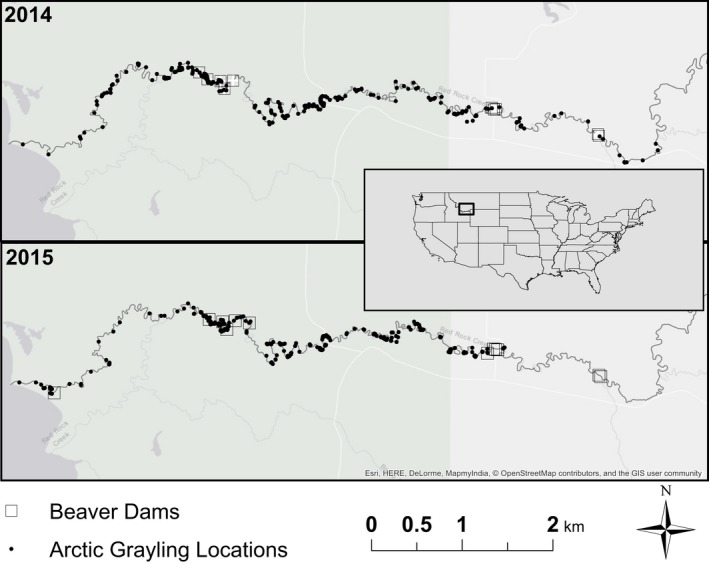
Map of the study area (Red Rock Creek, Red Rock Lakes National Wildlife Refuge, Montana, USA) with beaver dam and all fish locations collected throughout the study

Upper Red Rock Creek (44.61°N, −111.70°W, 2,017 m elevation, 110 km^2^; https://water.usgs.gov/osw/streamstats) is a cold, high‐mountain tributary of the Upper Missouri River in the eastern Centennial Valley of the Greater Yellowstone Ecosystem (Figure 2). The stream is characterized by a snowmelt‐dominated hydrograph with peak discharges (mean = 4.2 m^3^/s; range = 1.9–8.3 m^3^/s; data 1994–2017) occurring between 15 May and 2 July. Upper Red Rock Creek is a sinuous, meandering pool‐riffle stream that flows through a willow dominated floodplain supporting abundant populations of mammal, bird, amphibian, and fish species, including endemic Arctic grayling. Radiocarbon dating of beaver pond sediments (Persico & Meyer, 2013) and grayling phylogeographic patterns (Stamford & Taylor, 2004) indicates the two species have been sympatric in the Greater Yellowstone Ecosystem for over 8,000 years. While records from the early 1900s indicate large numbers of Arctic grayling were found in Centennial Valley streams and lakes, these populations experienced steep and persistent reductions in recent decades. Spawning historically occurred across at least twelve streams, but currently persists at low levels with actual spawning confirmed in only three streams. Managers believe Upper Red Rock Creek spawning supports the majority of the remaining adfluvial grayling population in the Upper Missouri River basin. During the non‐spawning seasons, adfluvial grayling swim downstream and reside in Upper Red Rock Lake, which is a shallow (<2 m) postglacial depression encompassing 893 ha where ice covers the lake for 7 months a year (Cutting et al., 2016).

### 2.2 Environmental conditions

Data from several remotely operated stations were used in our research, including daily air temperatures from a Remote Automated Weather Station site in the Centennial Valley maintained by the United States Forest Service (RRDM8, 44° 41' 00" N, 111° 50' 00" W, 2,039 m a.s.l.; https://mesowest.utah.edu), upper basin snowpack from an automated snow telemetry station at White Elephant, Idaho (44° 32' 00" N, 111° 25' 00" W, 2,350 m a.s.l.; https://wcc.sc.egov.usda.gov/nwcc/site?sitenum=860), and stream discharge from the Red Rock Creek gauging station operated cooperatively by the United States Geological Survey and Red Rock Lakes National Wildlife Refuge (44° 37' 01" N, 111° 39' 24" W, 2,033 m a.s.l.; https://waterdata.usgs.gov/nwis/uv?site_no=06006000).

### 2.3 Beaver dam characteristics

**Figure 3:**
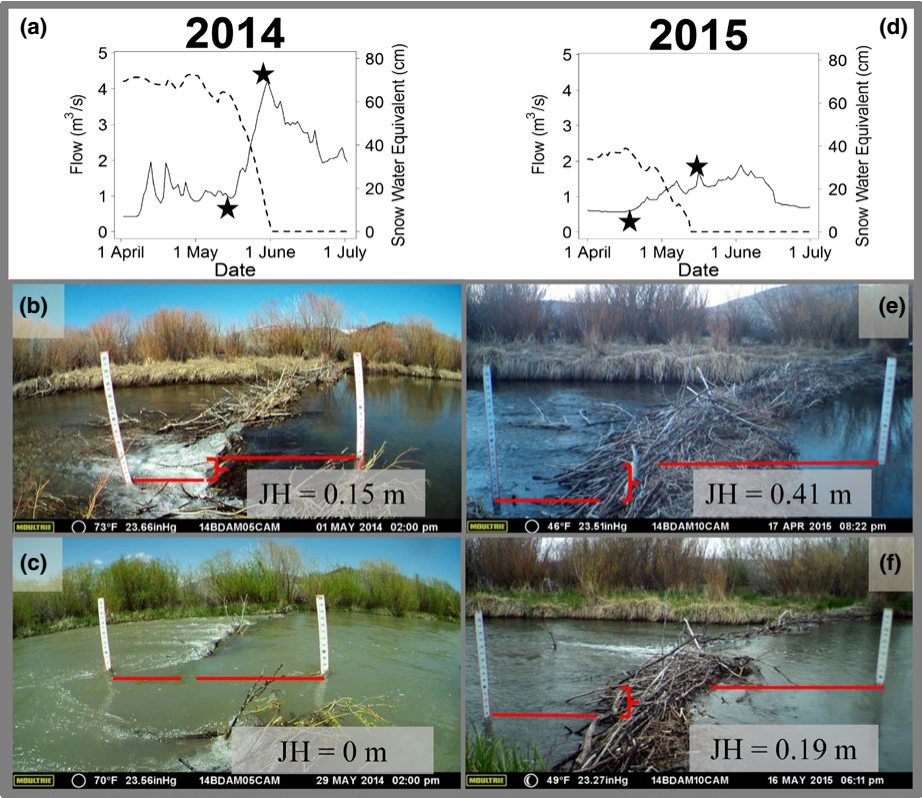
Within and across year comparison of the same dam in relation to jump height (JH). Jump height (m) distance was greater during low flows (panels b and e) than normal flows (panels c and f). Annual hydrograph shown for 2014 and 2015. Dam height for this same dam was greater in 2015 (0.96 m vertical height) than 2014 (0.78 m vertical height). Stars point to the daily flow on the day the photo was taken

Prior to spring snowmelt runoff, we inventoried beaver dam locations on Red Rock Creek from the lake confluence to the uppermost historic spawning extent of grayling (a total of 16 stream km) in both 2014 (*n* = 10 dams) and 2015 (*n* = 12 dams). We used a global positioning system (GPS) and photographs to geolocate all dams across both years of the study that spanned the entire creek width (*n* = 16 dams) or were breached (*n* = 6; Supporting Information Table S1, Supporting Information Figure S1). We define dams that did not span the width of the creek or were partially submerged as “breached” (Table 1). Two dams in 2014 and one dam in 2015 were breached throughout the season. In 2015, three dams became breached between 29 April and 7 May. We measured dam characteristics suspected to influence passage probability in spawning salmonids, including dam width, difference in height of upstream to downstream water level (hereafter, “jump height”; Figure 3), and difference in downstream dam water surface and bottom of creek channel (hereafter, “scour pool depth”; Supporting Information Table S1). We measured dam width along the longitudinal axis (i.e., with the flow of the downstream water) by averaging three evenly spaced locations from the point where the sticks ended on the downstream side of the dam to the upstream side where streambed sediments intersect the exposed sticks of the dam. We determined the number of hydrologic linkages, defined as natural flowing pathways around one or either side of the dam that connects sections of stream above and below the dam with ≥5 cm of water depth. We recorded links as a categorical variable: 0 (absent), 1 (present in only one side of the adjacent upland), or 2 (present on both sides of the adjacent upland). Additionally, maximum flooded area was measured around each dam at, or just after, high flow, as determined by existing water boundaries plus flood indicators such as sediment lines and flood debris. We digitized field collected GPS points and hand‐drawn maps on satellite imagery to create polygons of flooded area (ha; Table 1). To determine jump height, we installed time‐lapse cameras (Moultrie models 180i and M‐1100i) that took photos hourly from 19 April to 22 June 2014, and 15 April to 18 June 2015 to quantify the relative change in water levels between paired staff gauges upstream and downstream of all dams (Figure 3).

**Table 1:** Predictor variables for models of Arctic grayling passage and movement rates across in‐stream beaver dams

Model	Category	Predictor	Description	Type
Passage	Environmental‐level	Maximum flow^a^	Maximum stream discharge (m^3^/s)	Time varying
		Temperature^a^	Average daily air temperature (°C)	Time varying
	Dam‐level	Breached	Dam missing section of material or not	Time varying
		Dam distance from lake	Stream distance from lake origin (m)	Static
		Dam width	Longitudinal axis measurement of dam (m)	Static
		Flooded area	Maximum flooded area around a dam (ha)	Static
		Hydrologic linkages	Number of passable linkages ≥5 cm deep on either side of dam	Static
		Jump height^a^	Difference between up versus downstream water level (m)	Time varying
		Maximum scour pool depth	Difference in water surface to bottom of stream (m)	Static
	Fish‐level	Condition	Fish condition at time of capture	Static
		Length	Fish length (mm)	Static
		Sex	Male or female	Static
Movement	Environmental‐level	Maximum flow^a^	Maximum stream discharge (m^3^/s)	Time varying
		Temperature^a^	Average daily air temperature (^o^C)	Time varying
	Dam‐level	Distance to next upstream dam	Distance to next upstream dam (m)	Time varying
		Number of dams passed^a^	Number of dams passed	Time varying
	Fish‐level	Direction of movement	Whether a fish was moving up or downstream	Time varying
		Distance from lake	Stream distance from lake origin (m)	Time varying
		Length	Fish length (mm)	Static
		Sex	Male or female	Static

a Measurement taken across an observational interval of fish relocations.

### 2.4 Tracking fish movement

A sample of adult grayling from Upper Red Rock Lake was captured, measured, weighed and identified to sex, and implanted with radio transmitters fitted with mortality sensors and external antennas (Lotek MCFT2–3BM, 11 × 43 mm, Newmarket, ON, Canada) in September 2013 (*n* = 49 fish) and September 2014 (*n* = 49 fish) for a winter habitat and survival study of Upper Red Rock Lake (Davis, 2016). We subsequently tracked individual grayling moving through Red Rock Creek from 22 April until 18 June in 2014 (*n* = 35 individual fish), and from 20 April until 18 June in 2015 (*n* = 38 individual fish). We located fish using handheld telemetry units (Lotek SRX 400A receiver with antennae) operated by 1–2 field personnel** **who each walked sections of the stream from Upper Red Rock Lake to above the most upstream dam site spanning approximately 16 km of stream. The entire length of the creek was surveyed three times a week in 2014 and 2015. However, as fish reached their terminus upstream spawning location, we reduced sampling frequency to 1–2 times per week. All capture and handling were done in accordance with protocols approved by Montana State University Institutional Animal Care and Use Committee (2013–18) and Montana Fish, Wildlife, and Parks (49‐2014).

### 2.5 Modeling fish passage probabilities

Passage probability is based on the assumption that if a dam is not a barrier then grayling will pass through, presumably to access additional upstream spawning sites. We used a generalized linear model (GLM) with binomial distributed errors and a logit link function to model passage probability among categories of variables related to environmental, dam, and fish characteristics (Table 1). We recorded a total of 247 passage events and 86 barrier events across the two years of study (2014: passage events =126, barrier events =41, and 2015: passage events =121, barrier events =45). We assessed the probability of grayling passage of dams as related to (a) average air temperature, (b) average jump height, (c) dam width, (d) dam breach status, (e) hydrologic linkages, (f) distance upstream from the lake, (g) maximum scour pool depth, (h) maximum daily streamflow, (i) total extent of flooded area in the riparian zone for a given dam, (j) fish sex, (k) fish length, and (l) fish condition at time of capture during previous fall (Fulton's condition factor, Ricker, 1975; Table 1). We quantified passage failure at four spatial scales and found that predictive performance, using Matthew's correlation coefficient (Matthews, 1975) for the passage model, was maximized at scale 3, corresponding to six bends downstream from the dam (Supporting Information Appendix S1). We considered a dam as a barrier when a fish was found below the dam and was still below the same dam during the next relocation (days between relocation: 2014 = mean 2.71, range 1–6; and 2015 = mean 2.09, range 1–3) of that individual. In addition, we examined how effects differed between breached and unbreached dams by including an interaction between all predictors and the breached variable. We checked multicollinearity among variables and found low collinearity (all Pearson's *r *≤ 0.7). We used least absolute selection and shrinkage operator (LASSO) to perform model selection by reducing the number of potential predictor variables using the R package glmnet (Friedman, Hastie, & Tibshirani, 2010). We then obtained parameter estimates and standard errors from the best model using a GLM with standard maximum likelihood methods. Although it is known that model selection can introduce bias into parameter estimates when using maximum likelihood (Harrell, 2015), standard GLM's do allow us to estimate standard errors of the estimates. All variables were centered and scaled by their standard deviation so the magnitude of effect sizes describes the relative importance of the predictor variables on the scale of the linear predictors.

We then tested various random effect terms in the GLM including (a) a random intercept of fish ID (a unique label for each fish in the study), (b) determine whether passage events depend on the individual fish (outside of the individual‐level predictors of sex, length, and condition), and (c) test whether passage events at each dam are non‐independent by including dam ID (a label used to identify each dam in the study). We used Akaike information criterion (AIC; Burnham & Anderson, 2002) in program R 3.4.1 (R Core Development Team, 2018) to compare these random effect models to the standard GLM. All random effects models were fit with the lme4 package (Bates, Maechler, Bolker, & Walker, 2015).

After determining the passage probability for each dam from the best model, the cumulative probability of passing each dam was determined by multiplying the probability of passage for a given dam by the probability for all downstream dams. This allowed an examination of the compounding influence of multiple dams on fish passage. We calculated the cumulative passage probability using year‐specific averages of temperature and streamflow observed during the passage attempts. For the individual‐level predictors of fish length and fish condition, we used year‐specific averages. Finally, we evaluated cumulative passage probabilities separately for males and females to detect sex‐specific differences but found no difference between sex based on overlapping passage curves. To determine the 95% confidence intervals of the cumulative passage probability, we drew realizations of a fish passing through each dam using estimates and standard errors estimated from the best standard GLM model. We plotted relationships of passage for specific predictors by holding other variables constant at their median values.

### 2.6 Modeling fish daily movements

Past work has used time‐to‐event analysis (e.g., Castro‐Santos & Perry, 2012) to analyze the factors that influence the rate at which individual fish overcome potential barriers. The movement velocity analysis we conducted and time‐to‐event analysis both use the time required to cover a specific distance to model movement. However, the emphasis of the methods slightly differs. In a time‐to‐event analysis, we would be focusing on the factors that influence how long it takes a fish to pass a particular barrier, a useful approach for designing structures that enhance fish passage. In our movement velocity analysis, we instead focused on how dams affect fish movement relative to movement through open stream segments.

We tested the prediction that the average daily upstream velocity of grayling was reduced by (a) total number of dams passed within an observational interval and (b) distance to the next dam (Table 1) using a LASSO GLM with a Gaussian link to model changes in individual fish velocity. Velocity was calculated by dividing the distance traveled by the time interval between consecutive observations to obtain the average velocity. We added additional predictor variables to account for variation not directly related to our main predictions, but could be used to predict movement. These additional variables included (a) movement direction (up vs. downstream), (b) distance from lake, (c) maximum streamflow during the observation interval, (d) air temperature, (e) fish sex, and (f) fish length (Table 1). Finally, we were interested in upstream fish movement; we therefore removed movements that indicated fish were spawning or had completed spawning and moving downstream to their lake origin. Our criterion for removal occurred when fish did not move repeatedly for several observations before moving downstream and did not head back upstream before the end of the study, or when a fish moved downstream and did not head back upstream before the end of the study. This left us with 67 observations of upstream fish movement in 2014 and 16 observations of upstream fish movement in 2015. We used the model structure selected by LASSO to fit a standard GLM using maximum likelihood. We also fit a model with a random effect of fish ID, in order to account for potential systematic differences among fish that could not be explained by the individual predictors of fish length and sex.

## 3 RESULTS

### 3.1 Environmental and dam conditions

Distinctive differences in environmental conditions were evident between 2014 and 2015, including daily air temperatures (2014 = April: 1.3°C, May: 7.7°C, June: 10.6°C, and 2015 = April: 2.7°C, May: 7.1°C, June: 15.0°C), maximum snowpack (2014 = 72.4 cm, and 2015 = 38.6 cm), and maximum flow (2014 = 4.22 m^3^/s, and 2015 = 1.90 m^3^/s; Figure 3). There was a high degree of similarity between years in beaver dam locations (Figure 2) and structure across years (Supporting Information Table S1).

### 3.2 Factors influencing fish passage of beaver dams

We used the LASSO estimates to perform model selection and report parameter estimates from the GLM with the best model structure. We tested the fit of our selected model against mixed‐effects models with the same fixed effects and random intercept terms for both the dam and fish ID. We found that the random intercept terms for the dam ID (ΔAIC = 1.09) and fish ID (ΔAIC = 1.99) both performed slightly worse than the standard GLM, though the low ΔAIC values show that we did not have evidence that was strong enough to clearly distinguish between these models.

**Figure 4:**
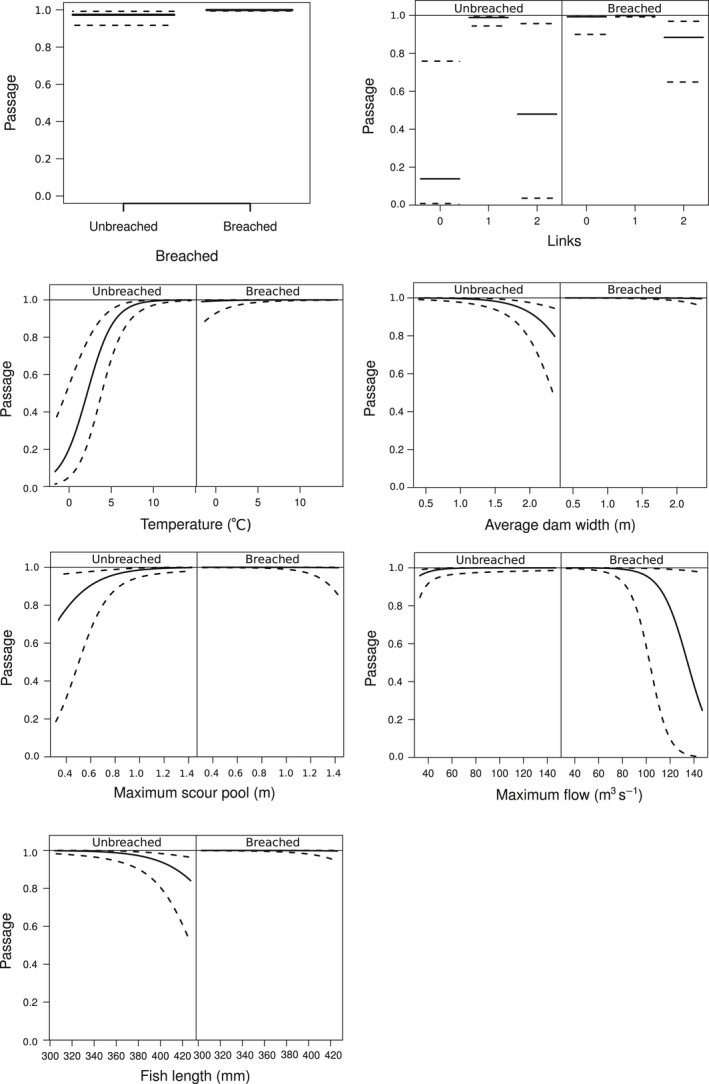
Predicted passage probability of Arctic grayling across beaver dams in relation to breached status, hydrologic links, temperature, dam width, maximum scour pool depth, maximum flow, and fish length for breached and unbreached dams. Dashed gray bands represent 95% confidence intervals associated with solid black lines showing mean passage probability for a given variable

We found that three predictor variables in our final model strongly influenced passage across dams (Table 2), including (a) breached status, (b) hydrologic links, and (c) temperature. Passage and barrier events remained consistent between years, but fish traveled significantly farther upstream on average in 2014 (Figure 2). The breached dam variable had a positive effect on passage probability as estimated by the GLM, along with air temperature and dams with one link (Figure 4, Table 2). Variables that had a moderate but positive effect on passage across unbreached dams include distance from the lake, maximum scour pool depth, and maximum flow (Figure 4, Table 2). Other variables that had a moderate but negative effect on passage across unbreached dams include dam width, no links, fish length, and fish condition (Figure 4, Table 2). We found negative interactions between the breached variable and temperature and between the breached variable and maximum flow. This suggests that increases in temperature and flow had a positive effect on passage at unbreached dams but had weak effects in breached dams. Finally, we found a positive interaction between scour pool depth and the breached variable such that passage was higher with increasing scour pool depths at breached dams than when a dam was unbreached (Figure 4).

**Table 2:** Parameter estimates for Arctic grayling passage and movement rate models across in‐stream beaver dams

Model	Category	Predictor	GLM mean (standard error)	*p*‐Value
Passage		Intercept	−2.74 (1.21)	0.024
	Environmental‐level	Maximum flow	1.28 (0.63)	0.041
		Temperature	2.94 (0.59)	<0.001
		Breached: maximum flow	−2.32 (0.74)	<0.001
		Breached: temperature	−2.01 (0.75)	<0.001
	Dam‐level	Dam distance from lake	2.09 (0.69)	0.0025
		Jump height	x	
		One hydrologic linkage	4.95 (1.50)	<0.001
		Two hydrologic linkages	0.46 (1.81)	0.8
		Dam width	−1.46 (0.42)	<0.001
		Maximum scour pool depth	1.18 (0.44)	0.0072
		Breached	6.84 (1.86)	<0.001
		Flooded area	x	
		Breached: one linkage	−1.61 (2.04)	0.43
		Breached: two linkages	−3.31 (2.21)	0.13
		Breached: maximum scour pool depth	2.56 (0.98)	0.009
	Fish‐level	Sex (male)	−0.51 (0.58)	0.38
		Length	−1.65 (0.55)	0.002
		Condition	−0.70 (0.35)	0.045
		Breached: length	0.87 (0.62)	0.16
Movement		Intercept	−0.30 (0.65)	0.65
	Environmental‐level	Maximum flow	0.081 (0.24)	0.74
		Temperature	0.21 (0.23)	0.37
	Dam‐level	Number of dams passed	−0.31 (0.22)	0.18
		Distance to next upstream dam	0.46 (0.23)	0.047
	Fish‐level	Direction of movement (upstream)	2.27 (0.64)	<0.001
		Distance from lake	0.93 (0.29)	0.002
		Sex (male)	0.37 (0.51)	0.48
		Length	0.12 (0.27)	0.66

x: the effect of a predictor variable was estimated as 0 using least absolute selection and shrinkage operator model selection.

### 3.3 Cumulative passage probability in relation to dams and distance from lake

**Figure 5:**
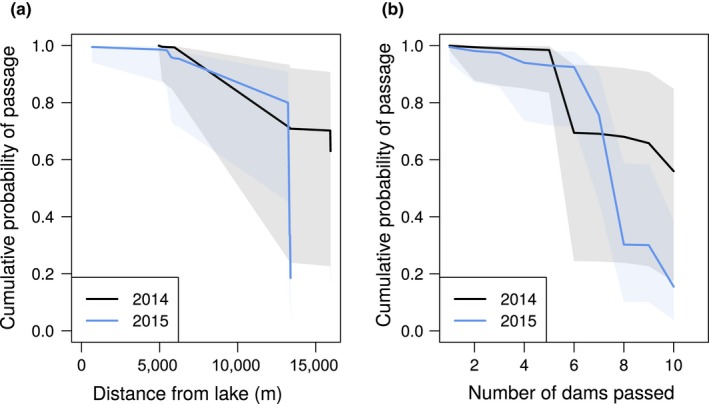
Across year variation in predicted cumulative passage probabilities as a function of (a) total distance (m) traveled and (b) number of dams passed during upstream migration. Shaded region represents 95% confidence interval, whereas the solid line shows mean effect

Cumulative probability of passage in the low flow year (2015) through the uppermost dam was less than 20%, with the largest bottlenecks being dam 9‐2015 (estimated probability of passage at dam: 44.4%) and dam 11‐2015 (56.9%) (Figure 5). Whereas in the normal flow year (2014), the largest bottleneck occurred again at dam 9‐2014 (70.6%) where cumulative passage probability was greater than 60% at the end of our study reach 15,976 m from the lake (Figure 5).

### 3.4 Influence of beaver dams on grayling movement velocity

**Figure 6:**
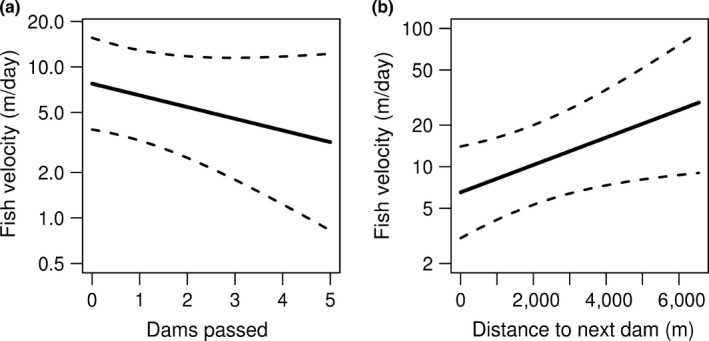
Predicted fish velocity (m/day) in relation to (a) number of total dams passed and (b) distance to the next dam (m). Dashed gray bands represent 95% confidence interval associated with black lines showing mean effect

Movement velocity rates of grayling decreased on average with increasing number of dams passed within a given observation (Figure 6, Table 2). Grayling also increased their average velocity the greater the distance to the next dam, further suggesting increased movement with unimpeded stretches of stream reaches (Figure 6). Additionally, grayling increased their movement rates when they were further from their lake origin. The following predictors had little effect on movement velocity, including (a) sex (male), (b) air temperature, (c) fish length, and (d) maximum flow. Our random effects model indicated that there were no system differences between individual fish outside of the predictor variables of fish length and sex (ΔAIC = 9.11).

## 4 DISCUSSION

The fundamental goal of our investigation was to understand the variables affecting upstream migration of an endemic population of Arctic grayling in relation to beaver dams and to determine how to maximize the success of the fish. It is important to note the average passage probability over unbreached dams, after controlling for other factors affecting passage at the dam level, was 88% (Figure 4), showing that Arctic grayling are clearly capable of navigating across beaver dams. We also found, however, that passage fell below 50% at specific dams. Our results reveal a set of controls on upstream movements of Arctic grayling that can be used by managers to predict, and potentially enhance, the migration success of Arctic grayling enroute to upstream spawning locations. We found that upstream passage of grayling was affected by three main characteristics: hydrology‐level effects including temperature, and dam‐level effects including breach status and hydrologic linkages.

In designing the study, we understood that the initiation of the spawning run for Arctic grayling on Red Rock Creek would be strongly controlled by stream temperature, as is the case generally with salmonids (e.g., Jensen, Heggberget, & Johnsen, 1986) and European grayling in particular (Ovidio, Parkinson, Sonny, & Philippart, 2004). In support of this hypothesis, grayling passage probability increased to >90% when air temperatures warmed between 6 and 10°C, while other conditions were average (Figure 4), providing additional support for the importance of temperature at initiating the motivation to pass dams while migrating upstream to their historic spawning areas. Wedekind and Kung (2010) observed similar temperature effects with increased passage of in‐stream structures by European grayling tied to water temperatures ranging from 6 to 10°C.

In addition to the importance of appropriate hydrologic conditions, the status of a dam (breached or intact) affected passage probability (Figure 4). However, we also found that breaching can interact with other factors leading to high impacts on fish passage. We found that increasing flows negatively affected passage for breached dams, but not unbreached dams, suggesting a possible velocity barrier where increased flow is being piped through the breached portion of the dam. Our results also reveal that individuals expected to be more fecund, given length‐ and condition‐specific fecundity relationships for grayling (Bishop, 1971), have lower passage across unbreached beaver dams.

Another important factor affecting the probability of fish passing unbreached dams is the presence of hydrologic linkages that provide a way for fish to swim around beaver dams (Figure 4). Our results show that one hydrologic linkage increases average fish passage probability by at least twofold compared to unbreached dams with zero and two links. It is important to note the high uncertainty around parameter estimates affecting the probability of fish passage across dams with zero and two hydrologic linkages, indicating other unmeasured variables at play such as reduced flow paths around dams. Beaver dams have the potential to force water out onto the floodplain through hydrologic linkages depending on the morphology of the surrounding landscape. As water is forced onto the floodplain, the total discharge and depth of flow in the channel is reduced (Levine & Meyer, 2014), leading to lower scour pools depths below dams. Indeed, dam metrics show that the lowest average scour pool depths are found at dams with two links while flooded areas are on average six times larger at dams with two links as compared to dams with one link. In line with this finding, shallower scour pools also negatively affected grayling passage. Similar results were shown by Kondratieff and Myrick (2006) for brook trout in an experiment where shallow scour pools (<10 cm) prevented fish (length >10 cm) from jumping waterfalls at least 43.5 cm, whereas when scour pools were deepened (>40 cm), all size classes cleared these vertical heights ranging from 63.5 to 73.5 cm. Future studies could better describe the effect of scour pool depths on fish passage through time using laboratory studies to determine the depth needed to provide a “leap pad” from a plunge pool; along with assessing how hydrologic linkages affect fish passage in terms of the level of inundation and how vegetation, depth, velocity, approachability, and location may affect how easily fish move through linkages.

For fish moving upstream in our study, intact dams with no and two hydrologic links negatively affected passage probability. Grayling are strong burst swimmers (Northcote, 1995) and may utilize localized hydrologic cues to navigate efficiently. When either no or two links are present, grayling may not receive strong cues to pass dams given that water is being diverted to the adjacent floodplain (Schlosser, 1995). On the contrary, when only one hydrologic link exists, flow is concentrated to a single side of the dam. This increased flow around one side of the dam likely attracts fish allowing them to find the passage. An alternative explanation is that when all the water in the stream is funneled into the one link, it makes a better pathway for upstream migration by creating a natural passageway that provides the flow and depth needed to “link” sections of stream.

We found little support for our prediction that jump height would negatively influence passage. Models predict a theoretical jump height for an average length grayling in our study (i.e., 37.5 cm) is 58 cm vertical distance based on the calculations presented in Reiser and Peacock (1985). Our measured jump heights for individual dams were less than the limits previously demonstrated for grayling for 19 of 22 dams monitored during our study, suggesting that jump height does not limit upstream passage, consistent with our empirical results. This result is supported by Jungwirth (1996) who showed European grayling passing vertical barriers up to 35 cm that separated several pools in a man‐made bypass channel around a hydroelectric dam. During our study, we observed fish passing dams by swimming through the lattice of willow branches, and it may be that jumping is not a common way for grayling to move past a dam. Additionally, in the normal water year (2014), most dams were completely inundated during peak flows corresponding with high‐mountain snowmelt, potentially allowing fish to more readily navigate past beaver dams (Figure 3c,f) as compared to low water years (Figure 3b,e).

We predicted that the probability of Arctic grayling passing dams would increase with normal to high flows and that cumulative fish passage would decrease as fish pass more dams during migration. Our analysis supports the prediction that the total distance traveled by fish was less in the low versus normal water year. We also found that the total number of dams passed in the low water year was less as compared to a normal water year (Figure 5), but given the wide confidence interval during the normal water year once six or more dams were passed, precluded us from identifying annual differences. Cumulative passage probability declined more quickly in the low water year as grayling migrated further up the creek in the normal water year, with no tagged grayling reaching their historic spawning area in the upper reaches of the creek during the low water year. Our findings of low cumulative passage probability support similar observations showing a strong relationship between stream discharge and passage rates of fish over beaver dams (Schlosser, 1995). In contrast, during the normal flow year, some fish passed all the dams in the study reaches. In addition, average cumulative passage probability exceeded 60% as grayling approached their historic spawning area 16 km from their lake origin. In both years, however, there was a high clustering of grayling locations for the middle reaches of the creek. It is possible that the grayling have shifted their spawning area to the middle reaches and are not migrating in large numbers to the historic spawning reaches 16 km from the lake due to delayed migration associated with barrier events occurring in lower sections of the stream, especially in years of low flows (e.g., 2014; Figure 3d–f). The potential shifting of their spawning area may impact current and future population viability, if the spawning habitat is of lower quality. Flemming and Reynolds (1991) showed that a delay in migration to spawning areas of grayling in Alaska resulted in a reduced distance traveled upstream and the subsequent selection of non‐preferred spawning habitats, likely leading to decreased recruitment.

We found support for the prediction that movement velocities increased in‐stream sections free of dams (Figure 5). The increasing velocity of fish associated with increasing distance to next upstream dam suggests that dams could be energetically costly and could delay arrival to spawning areas given velocities declined as grayling approached the next upstream dam. As documented in other salmonid species, arrival time to spawning grounds has been linked to mate acquisition (Quinn, Adkison, & Ward, 1996), increased fecundity and reproductive success (Dickerson, Brinck, Willson, Bentzen, & Quinn, 2005), and increased growth rates of young (Seamons, Bentzen, & Quinn, 2004). Slower movement velocities when approaching the next upstream dam potentially suggest that fewer individuals will arrive earlier, possibly indicating reduced reproductive output at the population level.

### 4.1 Conservation and management implications

Natural and artificial beaver dams have been observed to raise water table elevations (Bouwes, Weber, Jordan, Saunders, & Tattam, 2016), add cover for fish (Gurnell, 1998), increase habitat diversity (Levine & Meyer, 2014), and increase willow growth rates (Marshall, Hobbs, & Cooper, 2013). Beaver dams have also been used to help restore connectivity between rivers and floodplains, resulting in improved stream and streamside ecological integrity and function (Weber et al., 2017). Despite the potential ecological benefits of beaver in‐streams, there is uncertainty about the variables that affect fish passage of beaver dams in field‐based settings (Kemp et al., 2012). The lack of definitive answers becomes particularly acute when the fish species in question is rare and endemic, such as the Arctic grayling population in our study area of southwest Montana. The results of our models help resolve some of this knowledge gap by demonstrating there are specific dams under particular conditions that are having an outsized effect on passage probability. To ensure that our model results are robust, we strongly encourage future studies to test these results at new locations, particularly where it is possible to study paired streams with and without beaver dams.

It is important to note that 88% of all observations in our study resulted in a successful passage of an unbreached beaver dam. The high passage success suggests that most beaver dams on Red Rock Creek during the duration of the study pose little risk to passage for upstream migrating grayling. However, our results can inform management by identifying dams that may be potential barriers to upstream migrating grayling. The act of partially breaching, rather than fully removing a dam, allows the site to provide ecosystem benefits (e.g., Levine & Meyer, 2014, Pollock et al., 2014, Bouwes et al., 2016), even though some hydrologic benefits will be lost. In normal discharge years, management actions may not be needed when temperatures warm between 6 and 10°C during the time when grayling are actively migrating to their spawning areas, and all dams would be (a) located relatively far from the lake, (b) have one hydrologic linkage, (c) are <2 m wide, and (d) have deep scour pools on the downstream side. During normal discharge years, efforts could be made to avoid breaching many dams, but if there are concerns about a specific dam, or the fish population requires high spawning success, then dams with shallow scour pools and zero or two hydrologic links could be breached.

During low discharge years, breaching many dams may be necessary to increase fish passage. The decision about which dams to breach may be based on the hierarchy of variables discussed above. For example, with temperature being such a major driver for motivating fish to pass dams, cold spring temperatures (<6°C) that coincide with the initiation of spawning migration may necessitate breaching some dams, excluding those with one hydrologic link. Additional stream‐specific dam characteristics that should be considered for breaching after assessing hydrologic linkages include: (a) dams with shallow downstream scour pool depths (i.e., <65 cm), (b) dams near the lake (within <5,000 m), and (c) wide dams (i.e., >2 m wide). Managers facing pronounced low water situations where reproductive success is paramount, as is in our model species, should consider breaching dams with all of these characteristics.

The criteria developed through analyzing passage probabilities provide an alternative approach to making decisions that either subjectively remove‐all or leave‐all dams, a common practice among resource managers when it comes to managing beaver dams (Kemp et al., 2012). However, a weakness in our study design was how we characterized barrier events by using radio‐telemetry and manual detection walkover surveys along the stream. A combination of walkover surveys and stationary antennae placed at individual dams would have helped to more precisely characterize directed attempts to pass the dam resulting in a barrier event versus whether fish simply lacked the motivation to do so. We also assumed that grayling preferred the upper portions of the creek for spawning which is based on historic anecdote instead of objective redd survey data. Rather, spawning may occur in lower portions of the stream which allows for wider spatial distribution of spawning areas. Regardless, practitioners can still make more objective and transparent decisions through the use of these findings which is particularly important on public lands tasked with managing biodiversity and species of conservation concern. Even more importantly, managers can strive for ecological balance by maximizing the benefits of healthy, intact riparian habitats for entire communities of animals along with fish populations at risk of extinction by even one unsuccessful reproductive year.

## CONFLICT OF INTEREST

None declared.

## AUTHOR CONTRIBUTIONS

KAC and RL conceived the ideas and designed methodology; SD and KC collected the data; KC with input from KAC assembled the data files; JMF with input from KAC and MA analyzed the data; KAC, JMF, MA, and RL led the writing of the manuscript. All authors contributed critically to the drafts and gave final approval for publication.

## DATA ACCESSIBILITY

All grayling passage and movement data are available through the Dryad Digital Repository (https://doi.org/10.5061/dryad.70h0b6b).
