# Supplementary material for: Linking beaver dam affected flow dynamics to upstream passage of Arctic grayling
Source: Ecol Evol. 2018 Dec 4;8(24):12905–17. doi: 10.1002/ece3.4728 (PMC6308880; doi:10.1002/ece3.4728)
Supplement: Supplementary file 1 [file ECE3-8-12905-s001.docx]

**Figure S1.** Physical measurements made at each beaver dam. As described in the text, we measured at all beaver dams: maximum scour pool depth, jump height, dam height, dam width, hydrologic linkages and breached.**
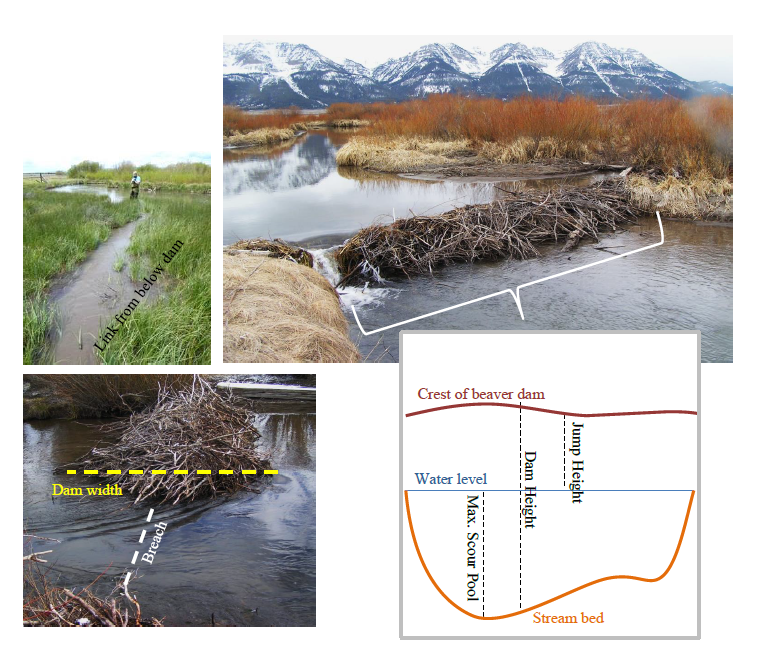
**
