# Supplementary material for: Linking beaver dam affected flow dynamics to upstream passage of Arctic grayling
Source: Ecol Evol. 2018 Dec 4;8(24):12905–17. doi: 10.1002/ece3.4728 (PMC6308880; doi:10.1002/ece3.4728)
Supplement: Supplementary file 2 [file ECE3-8-12905-s002.docx]

**Supporting Information**

**Table S1.** Table of dam characteristics for dams in 2014 and 2015 at Red Rock Creek, Red Rock Lakes National Wildlife Refuge, Montana, USA. Dams are ordered by distance to Upper Red Rock Lake. Dam names with same number indicate dam was present during both years of study, with specific year following the hyphen. We define dams with missing sections of material as “breached”. We measured dam characteristics we suspected might influence passage probability in spawning salmonids, including: dam width, dam height, difference in height of upstream to downstream water level (i.e., “jump height”), and difference in downstream dam water surface and bottom of creek channel (i.e., “scour pool depth”). We measured dam width along the longitudinal axis (i.e. with the flow of the downstream water) by averaging three evenly-spaced locations from the point where the sticks ended on the downstream side of the dam to the upstream side where streambed sediments intersect the exposed sticks of the dam. We determined the number of flooded bank links, defined as flooded areas on either or both sides of the dam with >5 cm of water depth. We recorded links as a categorical variable: 0 (absent), 1 (present in only one side of the adjacent upland), or 2 (present on both sides of the adjacent upland). Maximum flooded area was measured around each dam at, or just after, high flow, as determined by existing water boundaries plus flood indicators such as sediment lines and flood debris.

| **Dam Name** | **Year** | **Distance from Lake (m)** | **Breached** | **Links** | **Dam Width (m)** | **Jump Height (m; sd)** | **Dam Height (m)** | | | **Flooded Area (ha)** | **Max Scour Pool (m)** |
| --- | --- | --- | --- | --- | --- | --- | --- | --- | --- | --- | --- |
| 1 - 2015 | 2015 | 678 | 5/7/2015 | 0 | 1.85 | 0.08 (0.07) | | 1.54 | 0.00 | | 0.42 |
| 2 - 2014 | 2014 | 4948 | entire season | 1 | 2.36 | 0.35 (0.13) | | 1.29 | 0.03 | | 0.73 |
| 3 - 2014 | 2014 | 5162 | entire season | 2 | 1.63 | 0.67 (0.15) | | 1.36 | 0.03 | | 1.09 |
| 3 - 2015 | 2015 | 5162 | entire season | 0 | 1.93 | 0.77 (0.11) | | 2.58 | 0.00 | | 0.7 |
| 4 - 2014 | 2014 | 5466 | No | 1 | 1.27 | 0.36 (0.09) | | 1.14 | 0.13 | | 0.7 |
| 4 - 2015 | 2015 | 5466 | No | 1 | 1.20 | 0.36 (0.04) | | 1.13 | 0.07 | | 0.98 |
| 5 - 2014 | 2014 | 5755 | No | 1 | 1.45 | 0.02 (0.09) | | 1.06 | 0.04 | | 1.02 |
| 5 - 2015 | 2015 | 5755 | No | 1 | 2.00 | 0.35 (0.04) | | 1.58 | 0.11 | | 1 |
| 6 - 2014 | 2014 | 5960 | No | 0 | 0.95 | 0.34 (0.12) | | 1.14 | 0.00 | | 1.39 |
| 6 - 2015 | 2015 | 5960 | No | 1 | 1.43 | 0.44 (0.05) | | 1.68 | 0.10 | | 1.1 |
| 7 -2015 | 2015 | 6246 | 4/29/2015 | 0 | 1.67 | 0 (0.03) | | 0.93 | 0.00 | | 0.41 |
| 8 - 2015 | 2015 | 13236 | No | 0 | 1.77 | 0.06 (0.08) | | 0.91 | 0.00 | | 0.71 |
| 9 - 2014 | 2014 | 13336 | No | 2 | 1.69 | 0.21 (0.18) | | 0.78 | 0.35 | | 0.38 |
| 9 - 2015 | 2015 | 13336 | No | 2 | 1.55 | 0.27 (0.07) | | 0.96 | 0.34 | | 0.32 |
| 10 - 2014 | 2014 | 13361 | No | 1 | 1.55 | 0.12 (0.24) | | 0.85 | 0.24 | | 0.81 |
| 10 - 2015 | 2015 | 13361 | 5/2/2015 | 1 | 1.47 | 0.17 (0.08) | | 1.01 | 0.34 | | 0.67 |
| 11 - 2014 | 2014 | 13384 | No | 0 | 1.02 | 0 (0.03) | | 0.81 | 0.00 | | 0.72 |
| 11 - 2015 | 2015 | 13384 | No | 0 | 2.00 | 0.21 (0.07) | | 1.16 | 0.00 | | 0.27 |
| 12 - 2014 | 2014 | 15954 | No | 1 | 2.06 | 0.93 (0.08) | | 1.36 | 1.18 | | 0.49 |
| 12 - 2015 | 2015 | 15954 | No | 2 | 1.51 | 0.41 (0.03) | | 0.91 | 2.85 | | 0.46 |
| 13 - 2014 | 2014 | 15976 | No | 2 | 1.28 | 0.31 (0.42) | | 0.95 | 1.34 | | 0.39 |
| 13 - 2015 | 2015 | 15976 | No | 2 | 0.78 | 0.35 (0.03) | | 1.35 | 2.98 | | 0.17 |
