# Supplementary material for: Linking beaver dam affected flow dynamics to upstream passage of Arctic grayling
Source: Ecol Evol. 2018 Dec 4;8(24):12905–17. doi: 10.1002/ece3.4728 (PMC6308880; doi:10.1002/ece3.4728)
Supplement: Supplementary file 3 [file ECE3-8-12905-s003.docx]

**Appendix S1**

We quantified passage failure at four spatial scales to explore how to best model barrier events, since we did not know the proximity at which a “blocked” fish would occur at a dam. We used the Matthews correlation coefficient (Matthews 1975, hereafter “MCC”) to measure model performance of the generalized linear model (GLM) with a binomial distribution at each spatial scale. We assessed 4 spatial scales downstream from a given dam to define the barrier events; scale 1 = 2 bends downstream (111$\pm$ 69 stream m [mean $\pm$1 SD]), scale 2 = 4 bends downstream (270 $\pm$106 stream m), scale 3 = 6 bends downstream (404 $\pm$186 stream m), and scale 4 = 8 bends downstream (518 $\pm$251 stream m). We defined spatial scale as the number of bends in the stream based on research demonstrating European grayling use of pool-riffle sequences for pre- and post-spawning habitat (Ovidio et al. 2004). The predictive performance for the passage model was maximized at scale 3 (MCC=0.71), corresponding to 6 bends downstream from the dam. All scales had MCCs greater than 0.65, suggesting our results are robust to the specific scale that was used.
